# Evaluation of Different Interstimulus Rinse Protocols on Smoke Attribute Perception in Wildfire-Affected Wines

**DOI:** 10.3390/molecules26185444

**Published:** 2021-09-07

**Authors:** Jenna A. Fryer, Thomas S. Collins, Elizabeth Tomasino

**Affiliations:** 1 Department of Food Science and Technology, Oregon State University, Corvallis, OR 97331, USA; jenna.fryer@oregonstate.edu; 2 Viticulture and Enology Program, Washington State University, Richland, WA 99354, USA; tom.collins@wsu.edu

**Keywords:** smoke taint, wine, sensory analysis

## Abstract

Wildfires produce smoke that can carry organic compounds to a vineyard, which are then absorbed by the grape berry and result in wines with elevated levels of smoke-related phenols. These phenols have been found to have a large impact on the flavor of wines, being the cause of a smokey flavor with a lasting ashy aftertaste. When evaluating the sensory profile of these wines, there is an observed problem due to the lasting nature of these undesirable attributes and potential flavor carryover between samples. Through the use of standard and temporal attribute check-all-that-apply, this research desires to better understand the impact of smoke on the sensorial profiles of wines with various levels of smoke phenols (high, moderate, and low). Additionally, through the employment of different interstimulus protocols, the effectiveness of rinses on diminishing the smoke flavor in wines and optimal time separation were investigated. It was determined that a 1 g/L pectin rinse in between samples with a 120 s separation is optimal to ensure the removal of smoke attribute perception. This work also indicated the need to look deeper at the effects of the in-mouth hydrolysis of glyconjugate phenols that impact overall smoke flavor.

## 1. Introduction

With the increase in wildfire occurrence in wine regions around the globe, there are new challenges that winemakers face. Due to global climate change, it is estimated that elevated temperatures will increase the number of very high to extreme fire danger days by up to 70% by 2050, along with a lengthening of the wildfire season [1]. In 2021, the California wildfire season began in April, months before their traditional start, and will last through the grape growth and harvest season. California has had 17 of the most destructive fires occur in its history since 2000, with 6 of them occurring in 2020 alone. In the United States, the 2020 wildfires caused an estimated 8% reduction in grapes crushed nationwide and economic losses up to $3.7 billion [2].

The smoke from these wildfires carries organic compounds that are absorbed into the grape berry resulting in elevated levels of smoke phenols [3]. In wood fire smoke, there are over 500 volatile aroma compounds that wildfire smoke can contain and carry. The concentration of these compounds in the wine is heavily dependent on grape varietal, time in the growth cycle, duration of exposure, and environmental conditions, such as wind and topography, along with wine production practices [3,4]. These smoke volatiles then enter the grapes through the leaves or from direct absorption by the berries, with a higher concentration found in the skin than the pulp [1]. 

Smoke volatile phenols have been attributed to unfavorable sensory profiles in wine [5]. When grapes are exposed to wildfire smoke, the levels of smoke-related phenols are elevated in grapes and the resulting wine is described as smokey, dirty, and burnt with an ashy flavor lingering in the finish [6]. Guaiacol, 4-methylsyringol, and 4-methylguiacol have been predominantly used as markers of smoke taint in wine, due to being the most abundant phenols in woodfire smoke and their low sensory thresholds [5]. It has been found, however, that the use of solely these phenols as indicators of smoke taint may be flawed since reconstitution experiments using these phenols in concentrations found in smoke-affected wines is not representative of the sensorial experience [7,8]. Parker [4] defined the use of guaiacol, 4-methyl guaiacol, and 4-methylsyringol, along with *o*-, *m*-, and *p*-cresols as the optimal markers for smoke exposure. Other phenols, such as 4-ethylguaiacol, eugenol, 4-ethylphenol, and furfural, can contribute to the overall smoke flavor profile [8]. The aroma thresholds of these compounds have been primarily explored orthonasally, while their retronasal threshold research has been limited. It has been observed that retronasally the best-estimated threshold of guaiacol is 13 µg/L, 4-methylguaiacol is 65 µg/L, and syringol is 1650 µg/L in water [5]. These values may be different in wine systems, with research showing that higher concentrations are required for the orthonasal detection of these compounds in wine over water, along with different varieties of wine having differing thresholds [1]. Some of these phenols, predominantly guaiacol and 4-methylguiacol, can be introduced during winemaking through fermentation and aging in heavily toasted oak. The levels of these compounds can reach estimated threshold detection levels but do not lead to the same undesirable aroma and flavor profile, indicating the contribution of additional compounds not found in oak [9].

Along with free volatile phenols, the contribution of phenol glycoconjugates (phenols bound to sugars) has also been noted [10]. These bound phenols do not contribute to aroma but become unbound during fermentation and wine aging, where the free smoke compound can be perceived [11]. In addition, the bound smoke compounds may also become unbound in the mouth from interactions with salivary enzymes and bacteria [4,12]. It has been considered that the negative aroma attributes associated with smoke can be correlated to the volatile phenol composition, while the flavor attributes are most associated with the glycoconjugate phenols [13]. 

In sensory evaluation, carryover is an effect that must be taken into consideration when designing experimental procedures. Carryover is a sensorial bias that can occur during sensory evaluation caused by residual sensations of a sample influencing subsequent ones [14,15]. This phenomenon accounts for the inflation or deflation of ratings panelists give a sample based on the sample that precedes it [16]. This effect can take two forms: an assimilation effect or a contrast effect. The assimilation effect, also known as convergence, is when there is an observed increase in similarity between samples due to residual sensations causing the current sample to regress to similar properties to the previous [15]. In wine evaluation, this effect has been observed when evaluating astringency, due to the lasting drying sensation that can persist between samples, causing a cumulative perceived intensity increase from the first to last sample [17]. The contrast effect is when there is an observed increase in the perceived difference between samples [14]. This contrasting effect causes scores to be indicated as lower when preceded by a sample with a higher intensity level of the attribute, and vice versa [15]. The most traditional practice to mitigate these issues is based on proper experimental designs that are balanced for these effects, such as complete block designs, that spread this effect equally over all samples [15,16]. Another method that can be employed to further reduce this effect is the use of effective interstimulus protocols, consisting of effective rinsing and time separation. The purpose of rinsing, and other palate-cleansing procedures, are to maintain a baseline during sensory evaluation to ensure that there are no alterations to perception based on residual sensations [18].

In previous work on smoke impacted wines, there has not been consistency and consensus in time separation and rinses employed, ranging from pectin to mild acidic solutions with no forced rest between samples to over a minute separation [4,13,19,20]. This may have led to possible false positives or elevated recorded intensities of smoke-related attributes. Pectin rinses have been generally used in the analysis of wine attributes, including smoke-affected wines; however, no work has looked at the improvement of this rinse over water. The temporality of smoke attributes is an additional area with an observed effect, but limited research into the extent of this lingering sensation. Since there has been limited research into the practices that will best account for the unique nature of smoke-related attributes, the goal of this research is to determine the optimal interstimulus rinse protocol and time separation of samples during smoke attribute evaluation to mitigate any potential carryover effects. 

## 2. Results

Using Parker’s [4] definition of smoke markers in wine, the low, moderate, and high smoke phenol wines contained 2.92, 32.52, and 79.19 µg/L total smoke marker phenols, respectively (Table 1). The level of smoke marker phenols found in the low smoke sample is within limits that can naturally occur in certain grape varietals and is considered the control sample in further analysis. 

### 2.1. Study 1—Attribute Check-All-That-Apply (CATA)

The selected attributes were highly dependent on the sample (*p* < 0.001), with the high smoke phenol wine corresponding with smokey descriptors. From the symmetric plot (Figure S1), 100% of the variability was explained by F1 and F2, with F1 explaining 91.93% of the variability. Of the 18 attributes, red fruit, dried fruit, floral, smokey, woody, tobacco, ashy, ashy aftertaste, and burnt aftertaste all showed significance at a 90% confidence level. 

### 2.2. Study 2—Attribute Temporal Check-All-That-Apply (TCATA)

**Figure 1:**
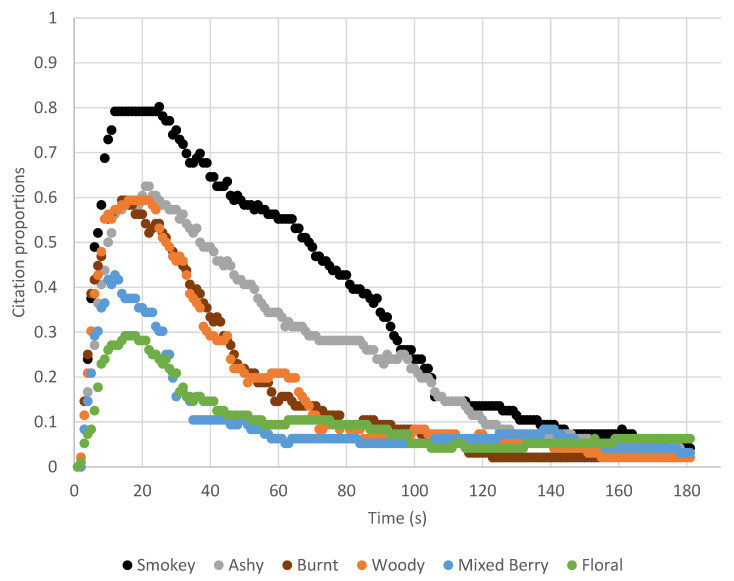
Temporality of TCATA attributes based on the proportion of overall citations in 1 s intervals over 180 s for the high smoke phenol wine.

From the TCATA analysis of the six attributes tested with a water rinse, it was found that the proportion of citations over all wines and attributes dropped below 0.1 and began to level out at approximately 120 s (Figure S2). Figure 1 shows how the temporality of each attribute varies, with smoke attributes eliciting a longer-lasting sensation.

### 2.3. Study 3—Fixed-Time-Point Intensity Evaluation (FTP)

**Figure 2:**
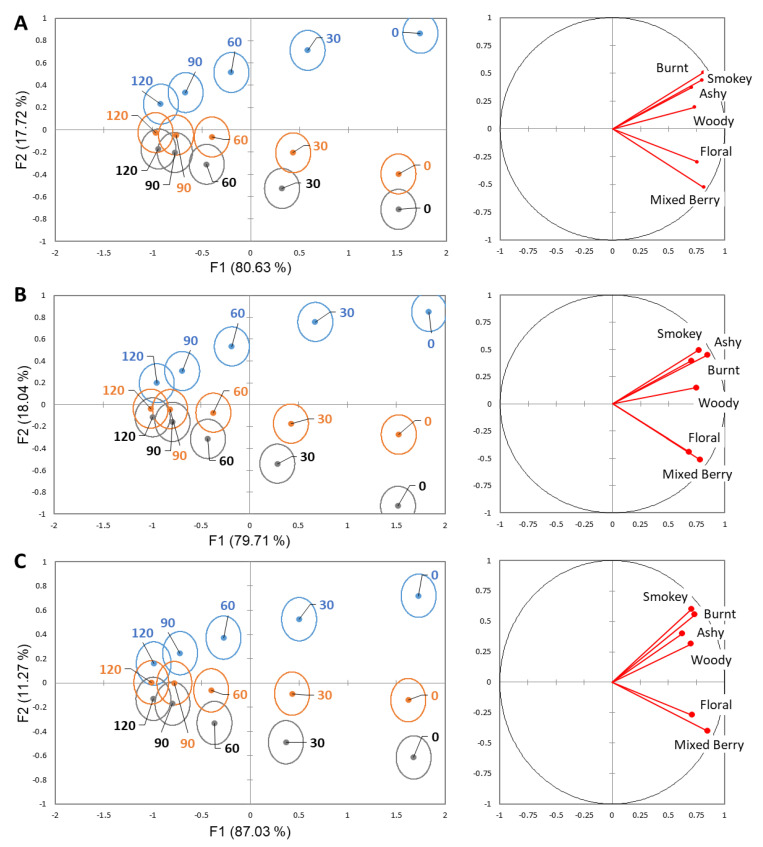
Separation of high smoke phenol (blue), moderate smoke phenol (orange), and low smoke phenol (grey) wines in 30 s intervals based on DA for each rinse system: (**A**) water, (**B**) pectin, and (**C**) mouthwash. Ellipses represent a 95% confidence interval, around the means.

Discriminant analysis (DA) showed how each rinse impacted the perception of the wines at 30 s intervals. A total of 97.75–98.35% of the variability is explained by F1 and F2 (Figure 2). F1 is strongly correlated with the time point, explaining 79.71–87.03% of the variability. F2 explains 11.27–18.04% of the variability, showing a relationship with the smoke phenol levels of the wines. Quadrant I shows a strong correlation with the smoke-related attributes, along with woody, while quadrant IV shows a correlation to the non-smoke attributes, floral and mixed berry. With the water rinse, the sensorial perception of the high smoke sample was significantly different than the low and moderate smoke phenol samples until 120 s, where it remained significantly different from the low smoke phenol sample (Figure 2A). For pectin rinse, all smoke phenol levels were significantly different until 60 s, where the low and moderate smoke phenol wines were no longer significantly different, and then at 120 s, when all wines were no longer significantly different (Figure 2B). For the mouthwash rinse, all samples were significantly different until 60 s, where the low and moderate smoke phenol wines were no longer significantly different, then 90 s, where the moderate and high smoke phenol wines were no longer different, and all wines were not significantly different at 120 s (Figure 2C).

**Figure 3:**
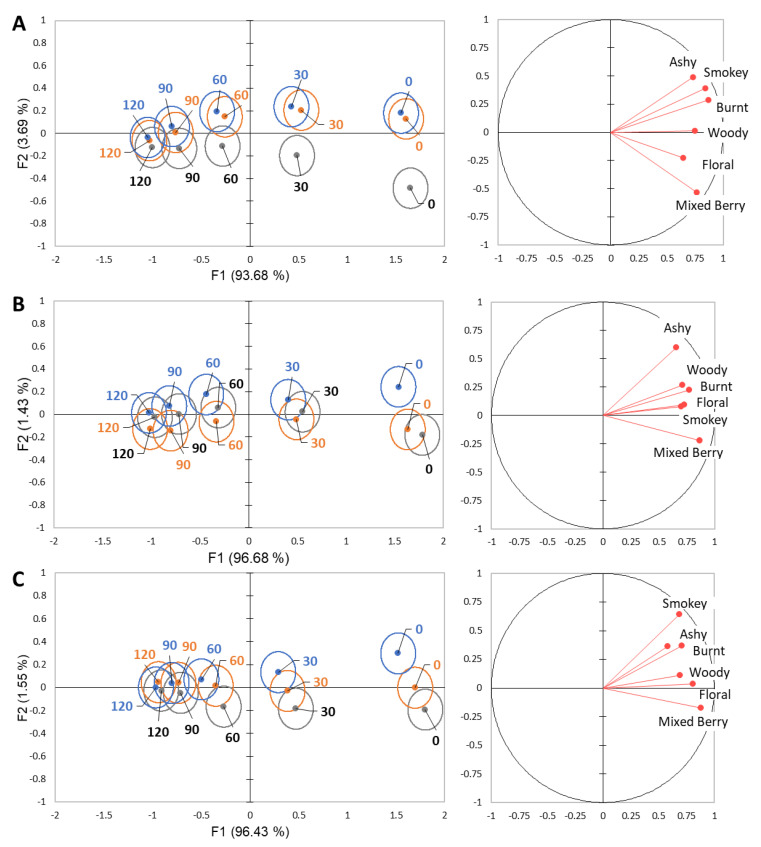
Separation of water (blue), pectin (orange), and mouthwash (grey) rinses in 30 s intervals using DA for each wine; (**A**) high smoke phenol, (**B**) moderate smoke phenol, (**C**) low smoke phenol. Ellipses represent a 95% confidence interval around the means.

By looking at the data separated by smoke phenol level rather than rinse, we can still see a high variability across the first two factors, 97.37–98.11% (Figure 3). As with the wine plots in Figure 1, the intensity of smokey attributes is strongly correlated with the time point, explaining 93.68–96.68% of the variability, while F2 is correlated to differences between the rinses, explaining 1.43–3.69% of the variability. In the high smoke phenol wine plot (Figure 3A), mouthwash showed significant differences from the other rinses at 0 s and 30 s. For the moderate smoke phenol wine, the water rinse showed a significant difference from the other rinses at 0 s (Figure 3B). For the low smoke phenol wine, water and mouthwash showed significant differences at 0 s and 30 s (Figure 3C). 

**Figure 4:**
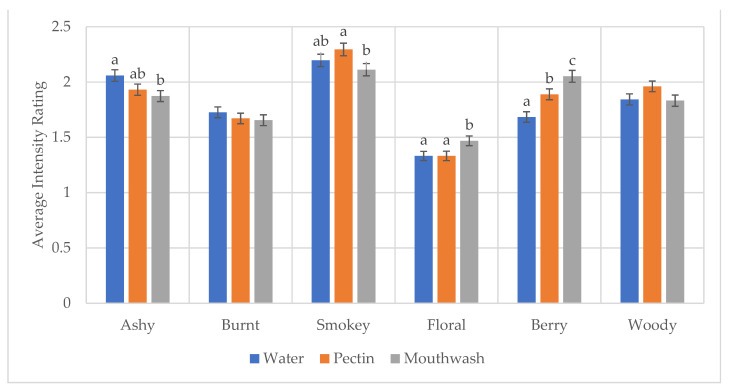
Average intensity over all wines and times for water (blue), pectin (orange), and mouthwash (grey). The same letters above bars indicate no statistical difference within each attribute as determined by Tukey HSD comparison of means at a 95% confidence level. Error bars represents standard error of the means.

When investigating attributes individually, resulting ANOVAs indicated that, across all attributes, wine and time showed significant differences (Table 1). The interaction of wine*time was also significant for all attributes except woody. The rinse variable was only significant for four of the six attributes evaluated (Figure 4). However, when looking at the interaction between rinse and wine, there was no significance for any attribute. An interaction of rinse*time was significant for mixed berry aroma but not for any other attributes, due to a perceived higher intensity of mixed berry for the mouthwash rinse at 0 s and 30 s (Figure S3). 

**Figure 5:**
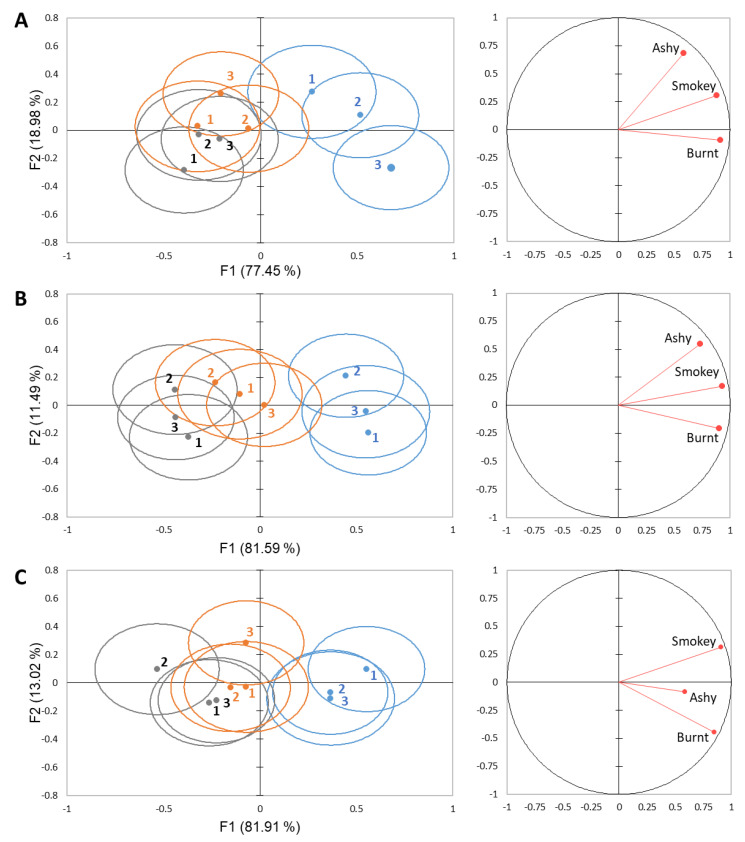
Separation of high smoke phenol (blue), moderate smoke phenol (orange), and low smoke phenol (grey) wines at each position within a set at 0 s based on DA from smoke-related attributes for each rinse system: (**A**) water, (**B**) pectin, and (**C**) mouthwash. Ellipses represent a 95% confidence interval, around the means.

To evaluate the effect of sample position within each set in study 3, DA using the smoke-related attributes was performed based on sample position and smoke phenol level at 0 s. This analysis indicated that 93.08–96.43% of the variability was explained by F1 and F2 when broken down by rinse (Figure 5). F1 shows correlation with the smoke phenol level of the wines, explaining 77.45–81.91% of the variability, while F2 shows correlation to the position, explaining 11.49–18.98% of the variability. For pectin and mouthwash rinses, there were no significant differences based on the position for each smoke phenol level (Figure 5B,C). For water rinse, there was a significant difference observed for the high smoke phenol wine when evaluated in set position 3 versus position 2 (Figure 5A). 

**Figure 6:**
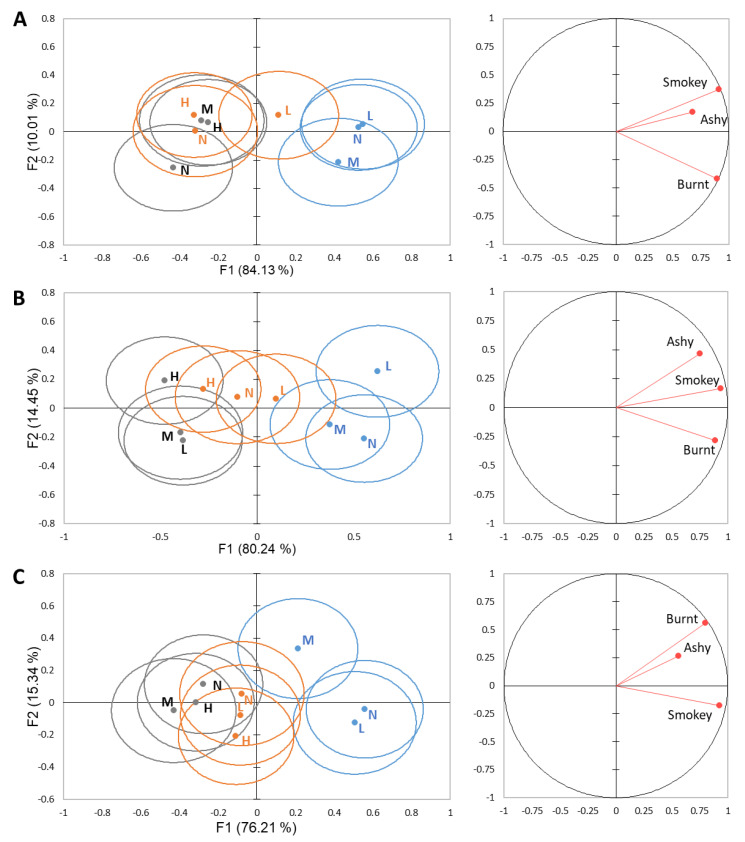
Separation of high smoke phenol (blue), moderate smoke phenol (orange), and low smoke phenol (grey) wines based on preceding sample (low smoke sample—L, moderate smoke sample—M, low smoke sample—L, and no sample—N) within a set at 0 s based on DA from smoke-related attributes for each rinse system: (**A**) water, (**B**) pectin, and (**C**) mouthwash. Ellipses represent a 95% confidence interval, around the means.

We also investigated if the level of smoke phenols in the preceding sample influenced the perception of the smoke-related attributes in the following sample using DA. The grouping factor in DA was preceding sample using attribute intensity data at time 0, showing that 91.54–94.69% of the variability was explained by F1 and F2. Similar to the analysis of set position, F1 shows correlation with smoke phenol level of the wines, explaining 76.21–84.13% of the variability, while F2 correlates to the preceding sample, explaining 10.01–15.34% of the variability (Figure 6). For each rinse, there are no significant differences observed based on the preceding sample for any of the wines.

## 3. Discussion

The alterations to the sensorial profiles of wine caused by smoke exposure were confirmed by these results. The wines in this study were produced from grapes with various levels of smoke exposure, and therefore smoke phenols, indicating that the wines would have different flavor characteristics. The low smoke sample contained levels of smoke phenols below detectable levels and within possible ranges for natural occurrence [8]. The levels of guaiacol observed in the moderately and high smoke phenol wines are greater than the detectable limits reported in previous literature [5]. Syringol and 4-methylguiacol were found in levels below observed detectable limits; however, the combination of these 6 marker phenols, along with others contribute to the total sensorial impact of smoke (Table S2) [5]. 

The smoke-related attributes, smokey, burnt, and ashy, were found predominantly and rated of higher intensity in the wines with elevated levels of smoke-related phenols [4,12,13]. Additionally, there has been observed masking of typical wine descriptors when smoke attributes are present [13]. This was further supported in our work, with mixed berry and floral flavor attributes having a lessened intensity in the high smoke phenol wine. The woody attribute was found to be more correlated with the smoke-related than the non-smoke attributes, which is most likely due to smoke phenols being found in certain woods, oak in particular. When looking at the TCATA results for attribute persistence over time, the smoke-related attributes had a dominating, longer-lasting presence. This confirms the lingering aftertaste that is described in previous work is different than the temporality of typical wine flavors [6]. It has been hypothesized that as residual wine remains in the oral cavity, there is continual hydrolysis of the glycoconjugate phenols that lead to lengthier perception [12]. This highlights the importance of a proper interstimulus rinse to remove as much residual wine as possible to not lead to increased breakdown and influence subsequent samples. From the DA results, there is clear a differentiation of the wines based on smoke phenol level. However, over time the sensorial perception of the wines became more similar and less differentiable, indicating flavor clearing from the mouth (Figure 2). 

For the three aspects investigated (wine, rinse, and time), rinse had the least impact on variability in DA, with wine and time contributing the majority of variability (Figure 3). This is then confirmed by the ANOVA, with the rinse variable showing significance in four of the six attributes and no significance being observed for any attributes when time and smoke phenol level are considered. To evaluate the effectiveness, the use of the high and moderate smoke phenol wines coming to statistical similarity with the low smoke phenol control from DA analysis was utilized. For water, in the 120 s period, the moderate smoke phenol wine reached statistical similarity with the low, but the high did not. This indicates water alone will require longer separation to ensure that a wine with a high level of smoke phenolics will not cause an assimilation carryover effect on the following sample. The pectin rinse, on the other hand, was effective in bringing the wines to statistical similarity. However, when looking at each attribute individually, pectin was only significantly different than water for the mixed berry attribute. In the analysis of the sensory profile of smoke-affected wines, pectin is the rinse predominantly used [4,12,21]. This work confirms that pectin shows improvement over water, when evaluating the attributes holistically, and is effective in clearing the mouth of residual smoke flavor with the 120 s of separation. In wine evaluation, pectin has been proven to be an optimal rinse due to its ability to complex with phenolic compounds [22]. Specifically, in regard to astringency, pectin interacts with tannins, forming a water-soluble complex that is then mostly removed with expectoration [17]. The use of pectin, however, does require an additional rinse with water before evaluating another next sample to ensure there are no residual sensations from small amounts of pectin solution remaining in the mouth.

Similar to pectin, the mouthwash pre-rinse brought all the wines to statistical similarity in 120 s. This rinse, though, showed other effects on the sensory profiles of these wines that are not desired when trying to understand the sensory impact of smoke. As observed in the DA plots, the use of this pre-rinse tended towards the non-smoke-related attributes, predominantly the floral and mixed berry flavors. This indicates a lessening of the masking effect observed with smoke flavor on these attributes. These results are consistent with the presumed effect of antimicrobial mouthwash on smoke phenols. With the use of a 0.2% chlorhexidine gluconate antimicrobial mouthwash, there was an inhibition of hydrolysis of the guaiacol glucoside for up to 2 hours post-application to an in vitro saliva and model wine mixture [12]. Chlorhexidine is a more powerful antimicrobial than the cetylpyridinium chloride used in this experimentation; however, these results show that even with this less potent solution there still is an effect on perception [23]. Although these results further indicate the potential breakdown of glycoconjugate phenols in the oral cavity and calls for further research, mouthwash was determined as ineffective for the understanding of the sensorial profiles of these wines. Not only did mouthwash effectively diminish smoke flavor post rinsing with water, but also altered the initial ratings (Figure 3). When determining the effects of smoke on wine, both from a research and industry standpoint, the initial ratings should be representative of the true, unmodified profile of the wine.

Regarding carryover, with 120 s separation between samples there was no evidence of alterations in smoke sensory profiles, within the evaluation sets, in all but one of the conditions and wines. The only observed effect was a significant difference between set position 1 and set position 3 of the high wine when evaluated with water. This difference was most likely caused by contrasting effects due to the strong differentiation that the high smoke phenol wine had from the other samples and panelist level of understanding of the attributes (Figure S4). To further mitigate these effects, greater panel training on the attributes can further ensure these contrast carryover effects are not present [15]. In evaluating the assimilation carryover effect, if this effect was present, the low smoke phenol and moderate smoke phenol wines would be more similar to the high phenol wine when preceded by a high phenol sample, indicated by higher intensity ratings. As seen in the DA plots, this was not present across all of the rinses (Figure 6). This lack of carryover may have not only been influenced by the time separation, but also the number of attributes being evaluated. The fewer the amount of attributes, the greater likelihood of carryover [15]. Since six attributes were evaluated, it can be assumed that if only a single “smoke” attribute was being evaluated, there may be an increased occurrence of carryover. It is therefore recommended that when evaluating smoke-affected wines to not just investigate a single attribute, such as smoke, but to include a mix of “smoke”-related attributes and desirable wine characteristics. Looking into the impact of set position with the use of pectin, there was the lowest amount of variability attributed to the set position when comparing pectin to the other rinse systems, further supporting the use of this rinse.

An additional concern for sensory analysis of smoke-affected wines that arose during this research is the high variation in smoke sensitivity between individuals. Six panelists had to be removed from the analysis of study 3 due to their clear lack of sensitivity to smoke-related attributes (no wines were rated as smokey) (data not shown). This further indicates the possible in-mouth effects on sensory perception and agrees with previous work [4,12,22]. In a study using a model wine containing guaiacol β-D-glucoside, it was found that there were individuals who were unable to perceive differences between a model wine containing the glucoside and the control [4]. Therefore, it is recommended that a panel screening procedure is used before evaluation. This ensures that all participants have some level of sensitivity to smoke-related compounds and that there will be no skewing of the data from individuals that do not perceive the smokey compounds.

## 4. Materials and Methods

### 4.1. Wine Samples

Field-planted Merlot grapevines at the Washington State University Roza research vineyard were exposed to simulated wildfire smoke during the 2017 and 2018 growing seasons. For the 2017 exposure, a mix of rangeland plants common in eastern Washington, including rabbit brush, big sagebrush, tumble mustard, and cheat grass, was used as the fuel source for smoke generation. For the 2018 exposures, softwood bark mulch was used as the fuel source. Sixty control (unsmoked) vines and sixty smoke-treated vines were enclosed in modular hoop-houses covered with 80/20 shade cloth for the duration of the 48-hour smoke exposure period. Both smoke-exposed and control fruit were harvested at commercial maturity and transported to the WSU Wine Science Center for processing. Wines were produced from both control and smoke-exposed fruit using a WSU standardized research wine-making protocol for red wines. Briefly, grapes were destemmed and crushed, then distributed to 200 L stainless steel fermenters which were filled to 120 L. The must was adjusted to a YAN level of 225 mg/L and inoculated with EC1118 yeast. Tank jackets were set to not exceed 30C and tanks were pumped over for 5 minutes every four hours. Wines were inoculated for the malolactic fermentation 48 hours after yeast inoculation. Wines were pressed after 10 days regardless of residual sugar level and SO_2_ was added after completion of malolactic fermentation, defined as malic acid concentration below 0.2 g/L.

Control and smoke-exposed wines from the 2018 smoke exposure trial were blended to create low, medium, and high smoke impact wines for this study. After preliminary sensory evaluation of these blends (data not shown), small (3.0–8.2% by volume) amounts of wines from the 2017 smoke exposure trials were added to the medium and high impact wines to adjust the differences in smoke impact across the set of wines for this study. It was necessary to use wines from 2017 as those had the higher smoke phenol levels compared to 2018 and were necessary to reach the concentration needed in the medium and high wines (Table 1 and Table S2). The sensorial impact of the smoke volatile phenols to be representative of high, moderate, and low intensity were confirmed with a preliminary tasting (data not shown).

### 4.2. Panelists and Standard Panel Procedures

Forty-three (13 M/30 F) (Study 1), forty-eight (17 M/31 F) (Study 2), and forty-six (17 M/19 F) (Study 3) panelists ages 21 to 60+ were recruited from the Oregon Wine Research Institute wine consumer database. Panelists were screened for being regular red wine drinkers, defined as consuming at minimum 1 serving of red wine per week. Exclusion criteria included smokers, women that are pregnant, individuals with taste deficits or other oral disorders, individuals with oral lesions or canker sores, individuals with tongue, lip, or cheek piercings, and individuals with wine allergies. All provided informed consent before participation and approval for work was granted by the Institutional Review Board at Oregon State University (IRB-8781). Study 1 and 2 encompassed a single evaluation session, while for study 3 each panelist participated in 1 training and 3 evaluation sessions.

All panels were completed in the Arbuthnot Dairy Lab on the OSU campus (Corvallis, OR, USA) and all tests were performed using Compusense Cloud Software® (Version 21.0.773.192939). A volume of 30 mL of each wine sample was served in black INAO wine glasses (Lehmann glass, Kiyasa Group, New York, NY, USA) and labeled with 3-digit codes. Panelists were provided with spit cups, distilled water, and unsalted saltine crackers (WinCo Foods Inc., Boise, ID, USA) at each session. To comply with university COVID guidelines, panelists evaluated samples in custom build tabletop booths (61 cm × 71 cm center, 61 cm × 65 cm sides) and two air purifiers (Winix, Vernon Hills, IL, USA) were used for air quality maintenance in the room. All booths were more than 7 feet apart. The temperature of the testing room was held at a constant 20–22 °C.

### 4.3. Study 1: CATA

Panelists were presented with all three wines in 3-digit coded glasses. They were instructed to sip and expectorate each sample. With focusing on the in-mouth experience and waiting 30 s to evaluate the aftertaste, they were instructed to check all attributes that applied to the sample from a provided list (Table 2). The terms chosen were based on previous research into smoke retronasal aroma profiles and typical flavor profiles of red wines [4,12,24]. In between each sample, there was a forced 1 min rest for panelists to rinse thoroughly with distilled water and, if desired, eat crackers. Attribute presentation order was randomized for each wine and wine order was randomized for each panelist. 

### 4.4. Study 2: TCATA

Based on the results from study 1, six attributes were chosen to be evaluated during Study 2 (Table 3). Before beginning formal evaluation, panelists were presented with a warm-up red wine (n.v. Merlot from California) and run through the TCATA evaluation system to prepare their palate for evaluation. Panelists were instructed to sip the sample, expectorate, and immediately start wine evaluation. After 10 s, they were instructed to rinse and spit with water. Over the course of three minutes, panelists were instructed to select and deselect attributes off a list as they perceived them. Three wines were presented at a time and each wine was evaluated in duplicate across two sets. There was a 1 min forced rest in between samples when panelists were instructed to rinse their palate with water. In between the sets, panelists were given an extended 5 min rest to cleanse their palate with water and, if desired, crackers. Attribute presentation order was randomized for each wine and wine order was randomized for each panelist. 

### 4.5. Study 3: FTP

#### 4.5.1. Rinses

In study 3, the use of three different interstimulus rinse protocols were employed. Rinses were provided in wine-rinse pairs and approximately 30 mL was given in 3-digit coded black glasses. For one week, panelists were presented with standard distilled water. Another week, the presented rinse was a 1 g/L pectin solution, prepared by suspending powdered pectin (Modernist Pantry, Eliot, ME, USA) in distilled water using an immersion blender (Mueller Austria Ultra-Stock, City of Industry, CA, USA). The third rinse was a two-part system where panelists, before beginning evaluation, were instructed to drink and swish 10 mL of dearomatized, concentrated cetylpyridinium chloride mouthwash (0.075%; Colgate-Palmolive Co, New York, NY, USA) for thirty seconds and then expectorate. Since the mouthwash was flavored, the rinse was boiled for 2 h and then reconstituted to 50% of the original volume with distilled water. During the evaluation, panelists were provided with 30 mL of distilled water in the wine-rinse pair. Rinse order was randomized for each panelist using incomplete block design, where each week approximately 33% of the panelist saw each rinse system.

#### 4.5.2. Training

Panelists participated in a single training session to be able to recognize the six chosen attributes, along with the system that would be used in the three formal evaluation sessions in study 3. The ashy, burnt, floral, mixed berry, and woody attributes were trained by panelists being asked to smell a reference and select the correct attribute off a multiple-choice list (Table 2). After selecting, they were informed of the identity and asked to re-smell the sample thinking of that attribute and what its experience would be like in-mouth since formal evaluation would be done retronasally. All aromas were then repeated. Order of multiple-choice options for each aroma and order of references was randomized for each panelist. For the smokey attribute, the three smoke levels of wine were used for training. Panelists were presented with each of the wines in 3-digit coded glasses and instructed to sip and expectorate each. They were then asked to select which sample they felt had the highest intensity of the smokey attribute. After selecting, they were informed of which sample had the highest intensity of smoke and asked to retaste this sample. This training was then repeated. The order of wines was randomized for each set.

For training on the fixed-time point evaluation system for study 3, panelists were instructed on the use of line scales and then was taken through the evaluation procedure with a training red wine (n.v. Merlot from California) paired with water in accordance with the procedure described in Section 4.5.3, twice. Finally, due to the unique nature of using the mouthwash and sensation in-mouth after rinsing, panelists performed a final training evaluation going through the mouthwash procedure before evaluating the training wine. After training, it was determined that the fixed-time point task required some clarification of instruction, and a video was provided for additional training at home on how time points would be prompted and evaluated (Video S1).

#### 4.5.3. Evaluation

**Figure 7:**
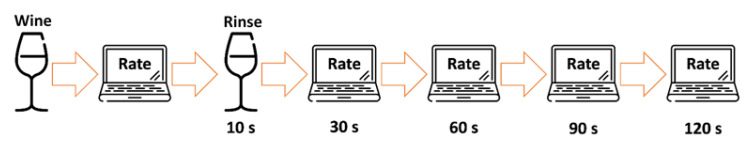
Flowchart of the fixed-time-point evaluation procedure employed in study 3.

Panelists were instructed to drink a sample of wine and then expectorate. As soon as they expectorated the sample, they pressed the green arrow to begin evaluation (Figure 7). Panelists immediately rated the intensities of all 6 attributes on an unstructured line scale, with “high” and “low” anchors at 90% and 10%, respectively. After 10 s, they were prompted to rinse and spit with the corresponding rinse. Every 30 s panelists were prompted to rate the intensities of the attributes at that moment. This continued for a total of 120 s for a total of 5 evaluation points (0 s, 30 s, 60 s, 90 s, 120 s).

Each week before beginning formal evaluation, after the mouthwash when applicable, panelists were presented with a warm-up wine (n.v. Merlot from California) and run through the evaluation procedure. After the warm-up, panelists were presented with all three wine-rinse pairs on a tray, and this was repeated for a total of three replicates. Between each wine-rinse pair, panelists were instructed to rinse with water. Between each set, there was a forced 1 min rest to cleanse the palate with water and, if desired, crackers. Attribute line-scale order was randomized for each wine and wine order was randomized within each set.

### 4.6. Data Analysis

All data analysis was performed using XLSTAT (XL Stat 2020.3.1 Sensory Package, Addinsoft, Paris, France) Correspondence analysis and CATA functions were used for study 1. TCATA analysis function was used for study 2, along with calculations of the proportion of overall citations in 1 s intervals. For study 3, the data for 6 panelists were chosen to be removed due to significantly lower intensity ratings of smoke attributes in the high smoke phenol sample. DA was performed, broken down by both wine and rinse systems, conducted at a 95% confidence level. Three-way ANOVAs using type III sum of squares analysis were performed for each attribute, with all interactions, for wine, rinse system, and time. Tukey HSD was used at a 95% confidence level for post hoc comparison of means. Discriminate analysis was also performed on effects of the sample set position by wine and preceding sample by wine at T = 0 s for each rinse system. 

## 5. Conclusion

From this experimentation, pectin was determined to be the best rinse employed for returning the mouth to baseline tasting conditions, indicated by statistical similarity of the moderate and high smoke phenol wines with the low smoke phenol control, along with no other sensorial impacts. The time separation between wines should be at least 120 s to ensure there are no carryover effects that augment the perception of smoke-related attributes above their true level. Although the pectin solution was the most effective in this analysis, 120 s is a lengthy separation to employ during both formal sensory evaluation and in-winery tastings to determine the extent of smoke impact. Other rinse systems should be studied to determine if there is an improvement in the amount of time required between samples. From considering the solubility of smoke-related phenols, this time may be able to be lessened for more efficient analysis. Mouthwash does show an effect on the sensorial profiles, which further indicates the possibility of oral breakdown of smoke glycoconjugates; however, mouthwash is not applicable for sensorial analysis of smoke attributes due to both effects on smoke attribute perception and alterations to other sensorial aspects. Based on this work, it is recommended a 1 g/L pectin rinse solution, followed by water, with 120 s of separation between samples should be employed for the sensory evaluation of smoke-affected wines.

## Figures and Tables

**Table 1:** Concentrations of smoke marker phenols in the three wines used for sensory analysis. All values reported in µg/L.

	Guaiacol	4-Methylguaiacol	4-Methylsyringol	*o*-Cresol	*m*-Cresol	*p*-Cresol
High Smoke	44.26	15.08	n.d	5.51	8.38	5.96
Moderate Smoke	16.52	3.83	n.d	2.87	4.41	4.89
Low Smoke	2.92	n.d	n.d	n.d	n.d	n.d

n.d indicates concentration is below quantification limits.

**Table 2:** Attributes evaluated during the CATA (Study 1) and TCATA (Study 2), along with standards used for training prior to FTPT (Study 3).

Study 1	Study 2/3	Aroma Standard
Red Fruit *	Mixed berry	Blended mixture of 46% frozen blackberries, 24% blackberry jam, 15% frozen strawberries, 15% cherry preserves
Dried Fruit *		
Dark Fruit	-	-
Floral *	Floral	3 drops of lilac and lilies with 3 drops violet essential oils dissolved in 1mL mineral oil
Black pepper	-	-
Smokey *	Smokey	-
Woody *	Woody	5 medium toast French oak wood chips
Leather	-	-
Earthy	-	-
Alcoholic	-	-
Tobacco *	-	-
Astringent	-	-
Chemical	-	-
Medicinal	-	-
Metallic	-	-
Ashy *	Ashy	1 tablespoon charred marshmallows ^1^
Ashy Aftertaste *		
Burnt Aftertaste *	Burnt	1 tablespoon burnt paper ash ^2^

* indicates significant *p*-value in study 1 from correspondence analysis (α = 0.1). ^1^ Mini marshmallows were spread on a baking sheet and baked in a 500 °F (260 °C) oven for 1 hour, then cooled and crumbled. ^2^ White printer paper was burnt to ash using a butane torch and let cool 24 hours in a closed jar.

**Table 3:** Summary of the significance of random effects of all attributes according to three-way ANOVA from fixed-time-point intensity ratings.

	Smoke Attributes			Non-Smoke Attributes		
	Ashy	Burnt	Smokey	Floral	Mixed Berry	Woody
Rinse	**	NS	*	*	***	NS
Time	***	***	***	***	***	***
Wine	***	***	***	***	***	***
Rinse*Time	NS	NS	NS	NS	*	NS
Wine*Time	***	***	***	***	***	NS

(NS) no significance, (*) significance, *p* < 0.05, (**) significance, *p* < 0.01, (***) significance, *p* < 0.001. Rinse*Wine and Rinse* Wine*Time not included as they were NS for all attributes

## Data Availability

Not applicable.

## Supplementary Materials

The following are available online. Figure S1: Symmetric plot from correspondence analysis of CATA selections for each wine; Figure S2: Proportion of overall citations (*n* = 1644) across all attributes over 180 s from TCATA analysis; Figure S3: Average mixed berry intensity over all wines at each evaluation time for water, pectin, and mouthwash; Figure S4: Separation of wines from agglomerative hierarchical clustering from CATA analysis; Table S1: Basic chemical analysis of three smoke levels of wine; Table S2: Concentration of non-marker smoke phenolic compounds measured in the three wines used for sensory analysis; Video S1: Fixed-time-point intensity evaluation system training.
