# Supplementary material for: Evaluation of Different Interstimulus Rinse Protocols on Smoke Attribute Perception in Wildfire-Affected Wines
Source: Molecules. 2021 Sep 7;26(18):5444. doi: 10.3390/molecules26185444 (PMC8470714; doi:10.3390/molecules26185444)
Supplement: Supplementary file 1 [file molecules-26-05444-s001.zip › Complete WB Images.pdf]

Supplementary Material: Prior Exposure to Coxsackievirus A21 Does Not Mitigate Oncolytic Therapeutic Efficacy

William J. Burnett, David M. Burnett, Gennie Parkman, Andrew Ramstead, Nico Contreras, William Gravley, Sheri L. Holmen, Matthew A. Williams and Matthew W. VanBrocklin

Figure 1A

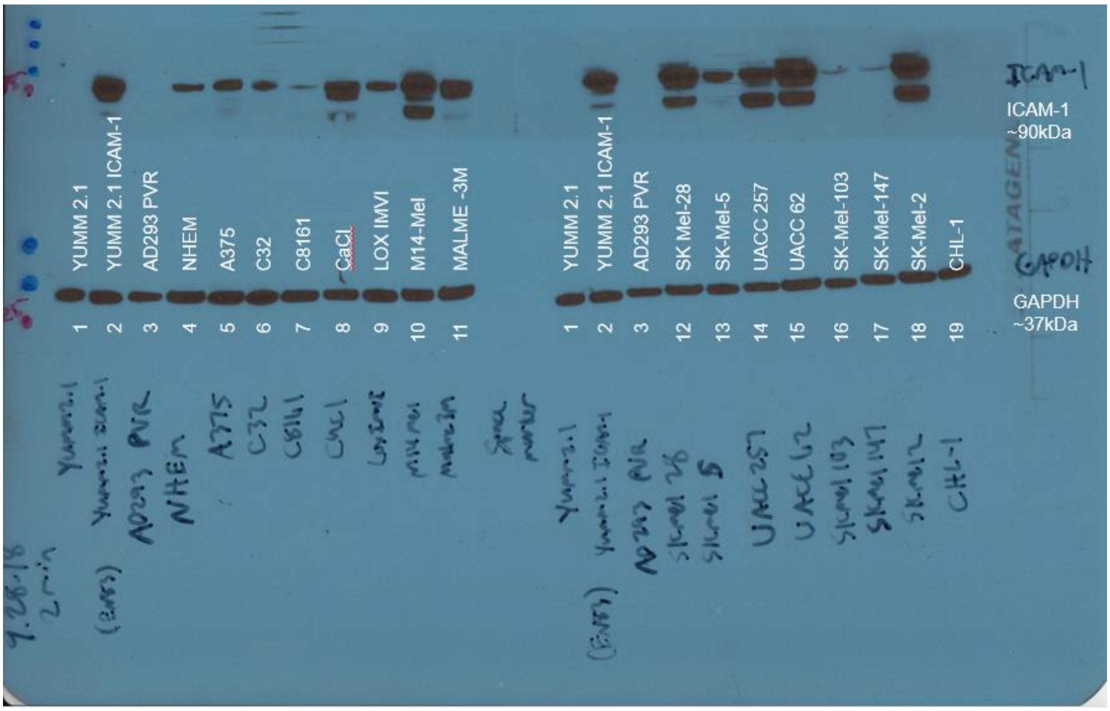

Figure 1B

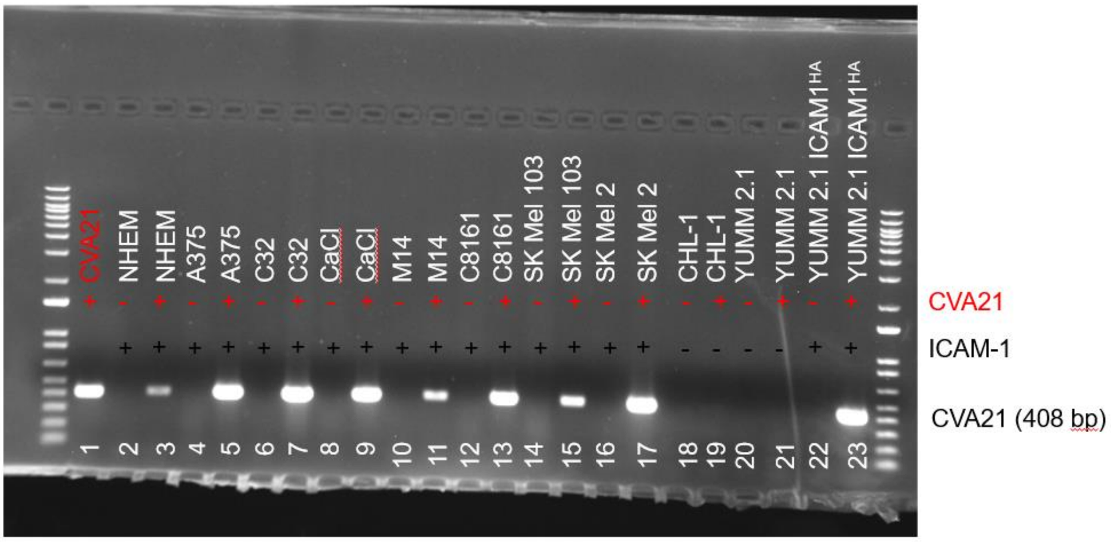

Figure 2A

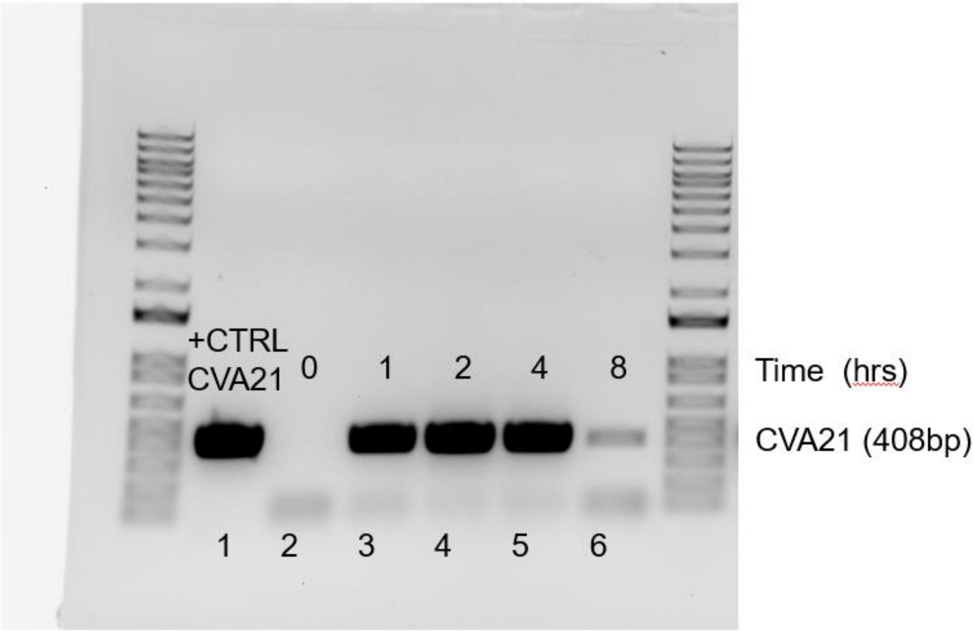

Figure 2B

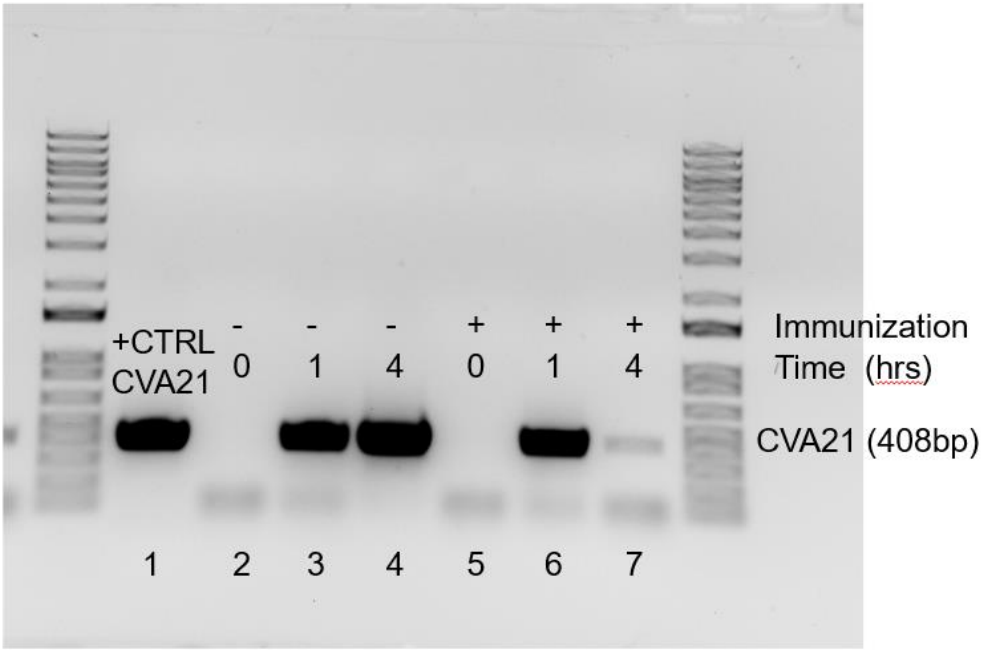

Figure 2D

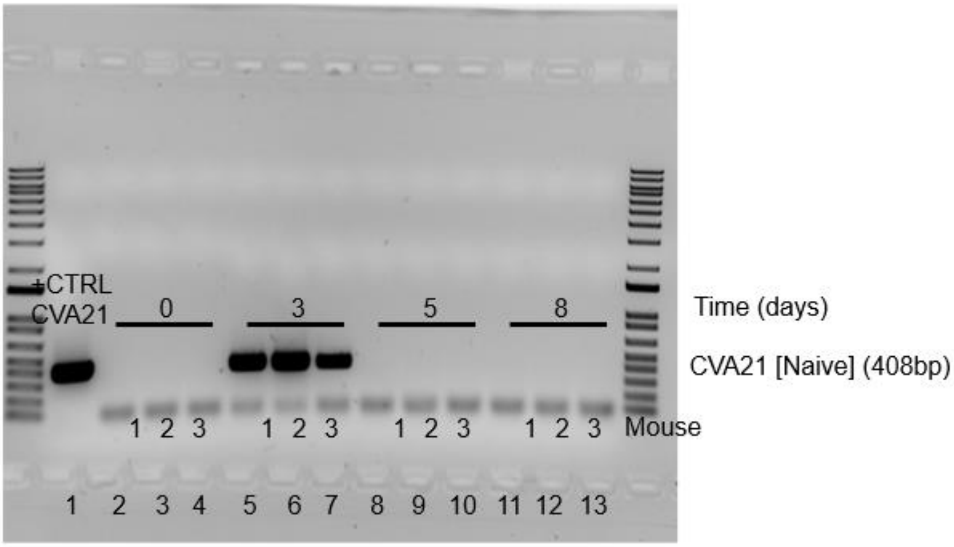
+CTRL
CVA21
0
3
5
8
Time (days)
CVA21 [Naive] (408bp)
1 2 3 1 2 3 1 2 3 1 2 3
Mouse
1 2 3 4 5 6 7 8 9 10 11 12 13

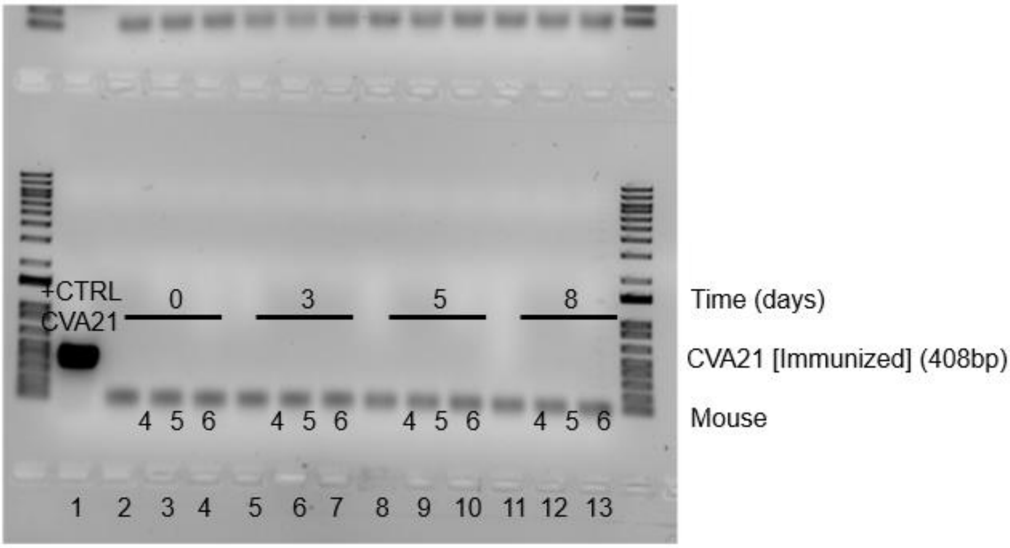
+CTRL
CVA21
0
3
5
8
Time (days)
CVA21 [Immunized] (408bp)
4 5 6 4 5 6 4 5 6 4 5 6
Mouse
1 2 3 4 5 6 7 8 9 10 11 12 13

Figure 3A

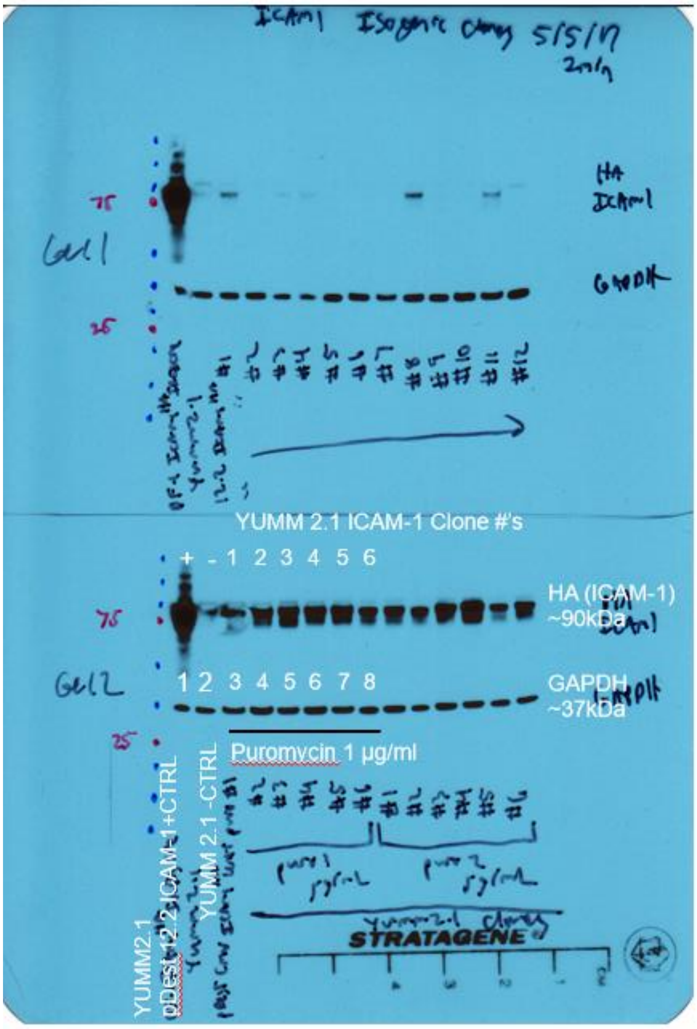

Figure 3B

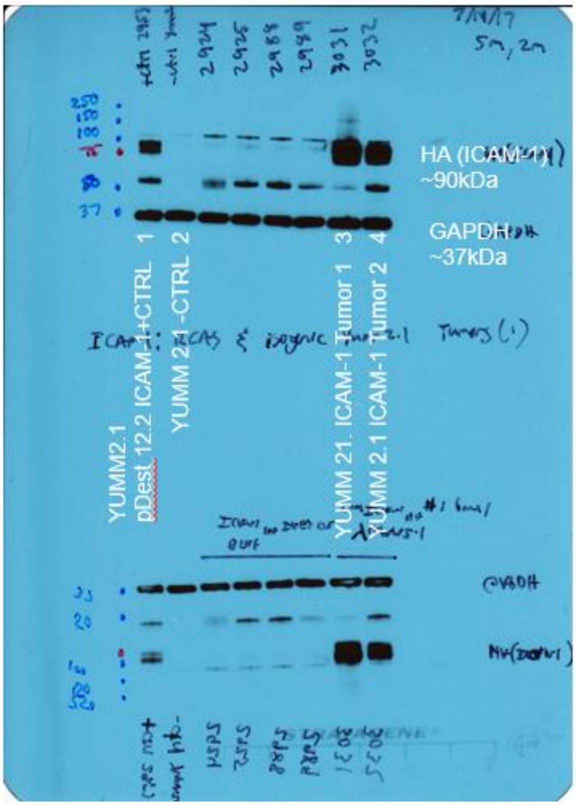

Publisher's Note: MDPI stays neutral with regard to jurisdictional claims in published maps and institutional affiliations.

© 2021 by the authors. Licensee MDPI, Basel, Switzerland. This article is an open access article distributed under the terms and conditions of the Creative Commons Attribution (CC BY) license (http://creativecommons.org/licenses/by/4.0/).
